# Metabolic Changes of Amino Acids and Flavonoids in Tea Plants in Response to Inorganic Phosphate Limitation

**DOI:** 10.3390/ijms19113683

**Published:** 2018-11-21

**Authors:** Santosh KC, Meiya Liu, Qunfeng Zhang, Kai Fan, Yuanzhi Shi, Jianyun Ruan

**Affiliations:** 1 Graduate School of the Chinese Academy of Agricultural Sciences (GSCAAS), Zhongguancun Nandajie, Haidian, Beijing 100081, China; kcsntsh@hotmail.com; 2 Tea Research Institute (TRICAAS), 9 Meiling South Road, Hangzhou 310008, China; liumeiya@tricaas.com (M.L.); hill@tricaas.com (Q.Z.); fankaitea@tricaas.com (K.F.); shiyz@mail.tricaas.com (Y.S.)

**Keywords:** *Camellia sinensis*, Inorganic phosphate, GC×GC-TOF/MS, UPLC-Q-TOF/MS, qRT-PCR, ICP-AES

## Abstract

The qualities of tea (*Camellia sinensis*) are not clearly understood in terms of integrated leading molecular regulatory network mechanisms behind inorganic phosphate (Pi) limitation. Thus, the present work aims to elucidate transcription factor-dependent responses of quality-related metabolites and the expression of genes to phosphate (P) starvation. The tea plant organs were subjected to metabolomics analysis by GC×GC-TOF/MS and UPLC-Q-TOF/MS along with transcription factors and 13 metabolic genes by qRT-PCR. We found P starvation upregulated *SPX2* and the change response of Pi is highly dependent on young shoots. This led to increased change in abundance of carbohydrates (fructose and glucose), amino acids in leaves (threonine and methionine), and root (phenylalanine, alanine, tryptophan, and tyrosine). Flavonoids and their glycosides accumulated in leaves and root exposed to P limitation was consistent with the upregulated expression of *anthocyanidin reductase* (EC 1.3.1.77), *leucoanthocyanidin dioxygenase* (EC 1.4.11.19) and *glycosyltransferases* (*UGT78D1*, *UGT78D2* and *UGT57L12*). Despite the similar kinetics and high correlation response of Pi and *SPX2* in young shoots, predominating theanine and other amino acids (serine, threonine, glutamate, valine, methionine, phenylalanine) and catechin (EGC, EGCG and CG) content displayed opposite changes in response to Pi limitation between Fengqing and Longjing-43 tea cultivars.

## 1. Introduction

Phosphorus is the second most limiting macronutrient for plant growth [1]. Inorganic phosphate (Pi) is a structural component of nucleic acids, phosphesters, and phospolipid. Pi has a vital role throughout cellular metabolisms and signal transduction cascades [2] that adjusts cell growth and development under specific nutritional conditions [3,4]. A complex array of morphological, physiological, and biochemical/metabolic adaptations to phosphorus starvation has been reported, coordinated to maximize internal Pi use and external Pi acquisition [5]. Morphologically, plants with deficient P supply increases root surface area and root:shoot ratios for exploration of more soil volume. Changes in global gene expression, protein levels, and metabolite contents under P deficiency have been extensively studied [6,7,8,9,10]. Metabolic profiling studies revealed that P limitation leads to a reduction of phosphorylated metabolites, but an increase in carbohydrates, organic acids, amino acids, and nitrogenous compounds [9,10,11,12,13]. Notably, the balance between synthesis and catabolic carbon metabolism is disturbed under Pi-stress conditions, and P limitation frequently leads to upregulated expression of genes and accumulation of secondary metabolites phenylpropanoids, flavonoid and their glycosides, and anthocyanin [10,14]. Previous studies revealed that gene expression, including signaling response and metabolism, was tremendously changed by Pi starvation [15]. Transcription factor *PHR1* is a major regulator of metabolic changes during P starvation [10]. The integral components of membrane *PHO1*, phosphate and the small molecule transporter from root to shoot can trigger Pi export in ectopic plant cells [16]. In *Arabidopsis*, *SPX*, which is involved in phosphate ion transporter and cellular response to phosphate starvation, was weakly induced [17]. Likewise, in rice *SPX2* was studied extensively in Pi sensing mechanism [18]. Previous studies have shown that there was a cross interaction effect between Pi deficiency and sugar signaling [19,20].

Tea (*Camellia sinensis*) is an important and widely consumed beverage globally. It is an important economic woody crop in Asian countries and P deficiency usually occurs in its growth environment, hence leading to a reduced production rate and deteriorated tea quality. The quality of tea, its taste and flavor are dependent on related constituents including amino acids and flavonoids, which are plant metabolites systematically regulated during growth and development of young shoots [21]. Furthermore, the metabolite flavonoids are additionally deemed to have health benefits to humans. Tea plants grow in acidic soil in which available P supply is usually inadequate due to low inherent content and strong fixation by abundant Al and Fe oxides. There have been several previous works concerning P treatment in tea plants [22,23,24,25,26]. P deficiency impairs photosynthetic electron transport chain capacity and deteriorates tea sensory quality by decreasing the concentrations of total polyphenols, flavonoids, total free amino acids, and specific metabolites asparatate, theanine (Thea,) and glutamic acid [22,23]. Ding et al. [24] conducted correlations between mineral elements and metabolites induced by P deficiency and P excess, finding that P stress reduces the syntheses of flavonoids and phosphorylated metabolites. As in other plants, P deficiency induces the accumulation of malate and citrate in the root and releases rhizosphere [25], which together with high P internal remobilization has been attributed to the tolerance mechanism of tea plants to low P supply [26]. Even though all these studies provided fundamental metabolite changes in response to different P statuses in tea plants, it remains unknown in terms of the molecular regulatory network behind P deficiency. Hence, our study tended to elucidate the transcription factor-dependent response of quality-related metabolites and expression profiles of genes to phosphate (P) starvation.

Teas of different types require variable characteristics of biochemical components in young shoots. For instance, young shoots suitable for green tea usually contain high free amino acids and relatively low catechins while those for black tea contain high contents of polyphenols and catechins. A selection of suitable varieties is a prerequisite to produce premium tea. In the present study, Fengqing Dayecha (*Camellia sinensis* var. *assamica*) which is suitable for black tea and Longjing-43 (*Camellia sinensis* var. *sinensis*) which is a good cultivar for green tea were chosen for P deficiency treatment. Untargeted metabolomics profiling based on GC×GC-TOF/MS and UPLC-Q-TOF/MS and in addition some targeted amino acid and catechins by High Performance Liquid Chromatography (HPLC) were used to investigate the change of metabolites in mature leaves, roots, and young shoots of tea plants to P starvation. To gain a deep understanding of the molecular mechanism, transcription factors and biosynthesis pathway genes were monitored in the meantime and collaboratively analyzed with metabolites. Our objectives were to gain systematical information on the response of quality-related metabolites (amino acids and flavonoids) and expression of genes to phosphorus starvation and genotypic variation of response among two cultivars suitable for different tea types.

## 2. Results

### 2.1. Biomass and Pi Concentrations in Plants

Tea plants of +P treatment contained higher concentrations of Pi (7.07 mg·g${}^{-1}$) than those of P-deficient (2.56 mg·g${}^{-1}$). We observed large decreases of Pi concentration of the whole plant in Fengqing and Longjing-43 under P starvation (by 51% and 71%, respectively) (Table 1). Biomass production was more depressed under P limitation (22.82 ± 7.1 g) than in P-sufficient conditions (28.46 ± 7.6 g), though the effect was not statistically significant (data not shown). The growth parameters of the root showed significant differences between the two P levels (Table 2) although there was no difference in biomass production (data not shown). Tea plants under P-starvation (−P) conditions had significantly decreased root volume, surface area, and number of root tips but increased root length and root thickness in comparison to those in sufficient (+P) treatment in Fengqing (Table 2).

### 2.2. Overview of Metabolomics Analysis of Tea Plants under −P and +P Conditions

Partial Least Squares Distinguished Analysis (PLS-DA) was performed and the model was established using a response sequencing test of 20 permutations of 5 components variables (−P and +P). The GC×GC-TOF/MS results showed that the Q2 was 0.97 and average cumulative R2 of X and Y of this model was 0.82, with 0.13 (R2) and −0.53 (Q2) values for intercepts on the Y-axis. In addition, UPLC-Q-TOF/MS showed a similar phenomenon with Q2 of 0.89. The average cumulative (R2) of X and Y for this model was 0.70, and the intercepts on the Y-axis were 0.23 (R2) and −0.54 (Q2), which indicated that the model was not over-fitting. To obtain a better regional difference and improve the effectiveness of the model and analytical ability, Orthogonality Partial Least Squares Distinguished Analysis (OPLS-DA) was performed. The GC×GC-TOF/MS model parameters of (R2X) and (R2Y) were 0.66 and 0.98 respectively and (Q2) was 0.98. This showed that a 66.7% variation explained 98.9% difference between the treatments. Likewise, UPLC-Q-TOF/MS model parameters of (R2X) and (R2Y) were 0.56 and 0.98 respectively and (Q2) was 0.95. Again, this showed that a 56.5% variation explained a 98.2% difference between the treatments. The predictive ability model of GC and UPLC were 82.8% and 77.35% and the difference values of (R2Y) and (Q2) (cum) was less than 0.4. However, (Q2) was greater than 0.5 that indicated a good predictive model to differentiate statistically cross verification of CV-ANOVA (*p* = 0.00) with significant treatment effect (Figure S1). The total number of profiled 39 primary and 36 secondary metabolites, where variable of importance in projection (VIP) > 1 were identified and selected as metabolites altering significantly based on GC×GC-TOF/MS and UPLC Q-TOF/MS respectively (Figure S1, Tables S1 and S2).

### 2.3. Metabolomics Changes in Primary and Secondary Metabolites

#### 2.3.1. Leaves

**Figure 1:**
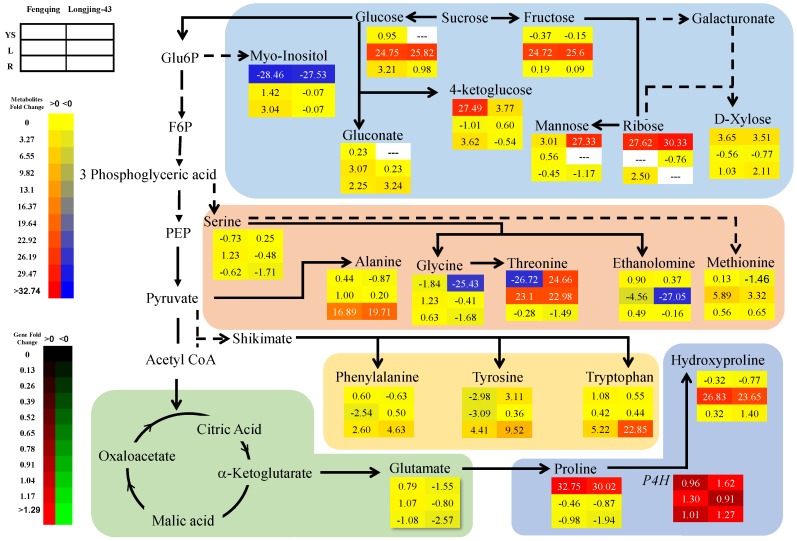
Fold change (log_2_^[−P/+P]^) of primary metabolites and *P4H*^a^ in response to P starvation. Fengqing (**left column**) and Longjing-43 (**right column**) and from the top to the bottom rows are young shoots (**first row**), leaves (**middle row**) and root (**button row**). YS, L and R represents young shoots, leaves, and root, respectively. Metabolites and metabolic genes inside background color box represents different biosynthesis pathway. The solid arrow shows direct and dotted arrow represents speculated steps in the pathway. The data depicted from Table S1 and Table 5, positive as increase and negative as decrease in fold change. On the false color scale red indicates increase; blue and green indicates decrease in metabolites and gene. ^a^*P4H* means *prolyl 4-hydroxylase*.

In mature leaves, the phosphatases containing compounds, i.e., d-mannose-1-phosphate, decreased immensely (>23-fold, log_2_^[−P/+P]^) in both cultivars (Figure 1 and Table S1). On the other hand, fructose (Fru) and glucose (Glc) in both cultivars highly accumulated (>24-fold) in mature leaves of −P treatment. However, gluconate also accumulated to a less extent (0.2–3.1-fold) in both cultivars of −P treatment (Table S1). Threonine (Thr) and hydroxyproline increased by 22.7–23.1 and 23.6–26.8-fold, respectively in two cultivars with −P treatment. By contrast, in −P treatment Phenylalanine (Phe) increase or decrease constantly (0.6-fold) in Fengqing and Longjing-43, respectively. Asparagine (Asp) showed a high-fold decrease in Longjing-43 (24.1-fold) only. P starvation led to marked increases of butanoic acid (21.5–26.8-fold), pyruvate (Pyr) (6.6–24.8-fold) in both cultivars and benzenebutanoic acid in Longjing-43 only (24.1-fold). On the other hand, pentanedioic acid decreased by 27.7–27.9-fold in −P treatment of two cultivars. Ethanolamine (EA) decreased in Longjing-43 (27.1-fold) and Fengqing (4.6-fold) by −P treatment.

**Figure 2:**
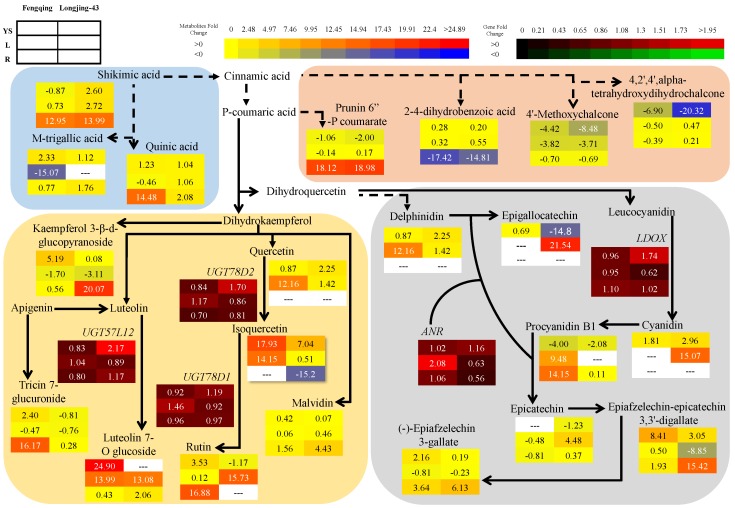
Fold change (log_2_^[−P/+P]^) of secondary metabolites and metabolic genes in response to P starvation. Fengqing (**left column**) and Longjing-43 (**right column**) and from the top to the bottom rows are young shoots (**first row**), leaves (**middle row**) and root (**button row**). YS, L and R represents young shoots, leaves, and root, respectively. Metabolites and metabolic genes inside background color box represents different biosynthesis pathway. The solid arrow shows direct and dotted arrow represents speculated steps in the pathway. The data depicted from Table S2 and Table 5, positive as increase and negative as decrease in fold change. On the false color scale red indicates increase; blue and green indicates decrease in metabolites and gene. *ANR*, *anthocyanin reductase*; *LDOX*, *leucoanthocyanidin dioxygenase*; *UGT57L12*, *flavanol 7-O glycosyltransferase*; *UGT78D2*, *flavanol 3-O-glucosyltransferase 2*; *UGT78D1*, *flavanol 3-O-glycoside L-rhamnosyl transferase 1*.

The response of secondary metabolites in mature leaves to P deficiency was largely variable among cultivars (Figure 2 and Table S2). P starvation increased luteolin-7-*O* glucoside (13- and 14-fold) and delphinidin (12.2- and 1.4-fold) in Fengqing and Longjing-43, respectively. A few flavonoids were specifically elevated by P limitation in cultivar, for example cyanidin (15.1-fold), epigallocatechin (EGC; 21.5-fold), rutin (15.7-fold) in Longjing-43, and isoquercitrin (14.2-fold) and procyanidin B1 (9.5-fold) in Fengqing. By contrast, P starvation led to decreases of 4${}^{\prime }$-methoxychalcone (3.7–3.8-fold), gallic acid (0.3–1.0-fold) in both cultivars, kaempferol-3-*O*-glucoside (6.2-fold) in Longjing-43, m-trigallic acid (15.1-fold), quercetin-3,4${}^{\prime }$-diglucoside (12.2-fold) in Fengqing.

#### 2.3.2. Root

The change of phosphates in root was less marked than that in mature leaves (Figure 1 and Table S1). P starvation led to a slight decrease of Frucose-6-phosphate (Fru6P; 0.2–1.4-fold), Glucose-6-P (Glc6P; 0.2–1.9-fold) and d-myo-inisitol-1 phosphate (0.6–1.7-fold) but increased d-erythro-Pentofuranose phosphate (0.4–0.7-fold) (Table S1). P deficiency moderately increased Glc (1–3.21-fold), d-xylose (1–2.1-fold) and gluconate (2.2–3.2-fold) in two cultivars, and ribose in Fengqing (2.5-fold). P starvation led to high increases of alanine (Ala) (16.9–20-fold), tryptophan (Trp) (5.2–22.8-fold) and tyrosine (Tyr) (4.4–9.5-fold) in two cultivars.

P starvation immensely increased quinic acid (2.1–14.5-fold), shikimic acid (13.0–14-fold), epiafzelechin-epicatechin 3,3${}^{\prime }$-digallate (1.9–15.4-fold), diosmetin 7-*O*-beta-d-glucuronopyranoside (13.2–17.8-fold), kaempferol 3-β-d-glucopyranoside (0.6–20.1-fold), tricin 7-glucuronide (0.3–16.2-fold) and prunin 6${}^{\prime \prime }$-p-coumarate (18.1–19.0-fold) but decreased 2,4-dihydroxybenzoic acid (14.8–17.4-fold) in two cultivars (Figure 2 and Table S2).

#### 2.3.3. Young Shoots

Phosphorus containing compounds in young shoots changed inconsistently among cultivars (Figure 1 and Table S1). Fru6P decreased in *cv*. *assamica* (Fengqing 30.9-fold) but increased in *cv*. *sinensis* (Longjing-43; 27.1-fold). Glc6P decreased in Fengqing (6.8-fold) but increased in Longjing-43 (5.5-fold). Myo-insitol-1-phosphate and erythro-pentofuranose phosphate increased in both cultivars and most remarkably in Fengqing. P starvation moderately increased carbohydrate d-xylose in Fengqing and Longjing-43 (3.5–3.6-fold), tremendously ribose (27.6 and 30.3-fold), and high mannose (27.3-fold) in Longjing-43. By contrast, P deficiency increased 4-ketoglucose by 27.5- and 3.8-fold and 2-ketoglutarate by 26.1- and 27.2-fold in Fengqing and Longjing-43, respectively. With P limitation, myo-inositol, derived from Glc6P, decreased in Fengqing (28.5-fold) and Longjing-43 (27.5-fold). Phosphorus deficiency caused significant changes of amino acids in young shoots. Thr was reduced in Fengqing (26.7-fold) but increased in Longjing-43 (24.7-fold) by Pi starvation. Phe was mildly decreased in Longjing-43 (0.6-fold) only. Remarkably, isoleucine (Ile) and proline (Pro) increased by 25.1–27.3-fold and 30.0–32.8-fold, whereas glycine (Gly) and leucine (Leu) decreased by 1.8–25.4 and 28.7–29.9 in both cultivars (Table S1).

P starvation significantly affected the accumulation of secondary metabolites (Figure 2 and Table S2). The uniformly increased secondary metabolites in both cultivars tremendously changed isoquercitrin (7.0–18.0-fold), epiafzelechin-epicatechin 3,3${}^{\prime }$-digallate (3.0–8.4-fold), quercetin 3-glucoside (2.0–4.3-fold), quercetin -(2${}^{\prime \prime }$-galloyl-α-l-arabinopyranoside) (3.1–15.6-fold), m-trigallic acid (1.1–2.3-fold) and those mildly changed delphinidin (0.9–2.2-fold), catechin (0.99–1.1-fold) and epigallocatechin gallate (EGCG, 0.9–1.0-fold). On the other hand, P starvation decreased procyanidin B1 (2.1–4), prunin 6${}^{\prime \prime }$-p-coumarate (1.1–2.0-fold), kaempferol 3-sophoroside 7-glucuronide (0.4–15.2-fold), quercetin-3-sulfate (0.3–1.4-fold) and chalcone (4${}^{\prime }$-methoxychalcone and 4,2${}^{\prime }$,4${}^{\prime }$,alpha-tetrahydroxydihydrochalcone) (6.9–20.3-fold). However, there were different responses among cultivars. For example, under low P supply, rutin, kaempferol-3-*O*-glucoside, (−)-Epigallocatechin 3,3${}^{\prime }$-di-gallate and catechol decreased in Longjing-43 but increased in Fengqing (Table S2).

**Figure 3:**
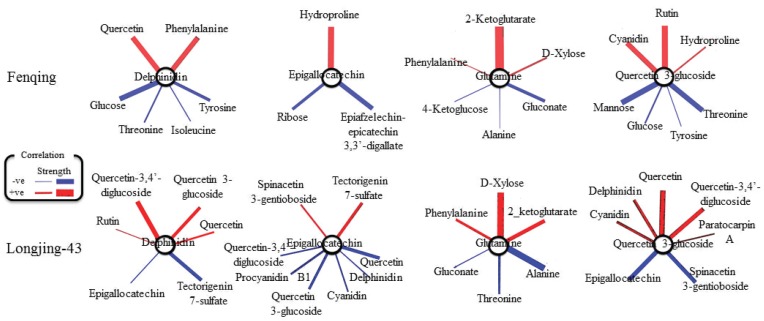
Correlations between fold change (log_2_^[−P/+P]^) of selected significantly changed (p< 0.05, *t*-test) metabolites in young shoots of Fengqing and Longjing-43. Red and blue colors indicate positive and negative coefficients and line thickness indicate correlation strength.

Due to their important role influencing tea quality, the fold changes of delphindin, EGC, glutamine (Gln) and quercetin-3-glucoside in young shoots were selected and subject to Pearson’s correlation analysis with other metabolites to screen the most related metabolites based on each cultivar (Figure 3). The results indicate that delphinidin which was mildly induced in Fengqing by P deficiency significantly and positively correlated with Phe but negatively with glucose (Glc), Thr, Tyr and Ile. Interestingly, there was significantly strong and negative correlation between EGC and epiafzelechin-epicatechin 3,3${}^{\prime }$-digallate. The secondary metabolites, quercetin 3-glucoside, have a significant and positive correlation with cyanidin and rutin. In addition, the major amino acid Gln had positive and almost similar fold change correlation with d-xylose and Phe but weakly and negatively correlated with Ala.

Young shoots of Longjing-43 and delphinidin had positive correlation with rutin and quercetin while negatively with EGC and tectorigenin 7-sulfate (Figure 3). EGC had strong and negative correlations with delphinidin, cyanidin, procyanidin B1, quercetin 3,4${}^{\prime }$-diglucoside and quercetin 3-glucoside. Likewise, quercetin 3-glucoside showed a strong and positive correlation with anthocyanin-related compounds (delphinidin, cyanidin, quercetin 3,4${}^{\prime }$ diglucoside and paratocarpin A). Gln showed positive correlation with d-xylose, Phe and 2-ketoglutarate and negative ones with gluconate, Thr, and Ala.

### 2.4. Targeted Analysis of Amino Acids and Catechins in Young Shoots

Young shoots are the harvestable plant organ for made tea production. The quality-related components, i.e., free amino acids and catechins in young shoots then were further quantified by targeted analysis (Table 3). Most of the free amino acid concentration in young shoots were significantly changed by −P treatment (Table 3). Asp, Gly and Leu were significantly decreased (19–76%) while Pro and Ile significantly increased (51–71%) by −P treatment in both cultivars. However, some other amino acids responded to −P treatment differently between the two cultivars. Glutamate, Val, Met and Phe increased (78–81%) in Fengqing but decreased in Longjing-43 (44–75%) whereas Thea, Serine (Ser) and Thr decreased in Fengqing but increased in Longjing-43 under P starvation. Arg and Tyr significantly increased only in Fengqing and Longjing-43, respectively by P deficiency. Other amino acids (His, Ala, Cys and Lys) was not affected by −P treatment. Fengqing contained a higher concentration of Glu than Longjing-43. The relative composition of free amino acids in Fengqing was changed, showing that Thea predominated in P-sufficient young shoots while Glu and Thea became the two major compound by −P treatment. In Longjing-43, Thea predominated in the pool of free amino acids regardless of P status. The concentrations of catechins in young shoots were affected by P treatment differently in the two cultivars. EGCG is the main catechin in young shoots and was affected (increase by 32%) by −P treatment only in Fengqing. EGC, ECG and GC increased (61–83%) in Fengqing but decreased (by 28 and 82%) in Longjing-43. Only catechin increased (62–70%) in both cultivars.

### 2.5. Response of Pi-Dependent Transcription Factors and Metabolic Genes to P Conditions

#### 2.5.1. Pi-Dependent Gene Transcriptional Expression

**Figure 4:**
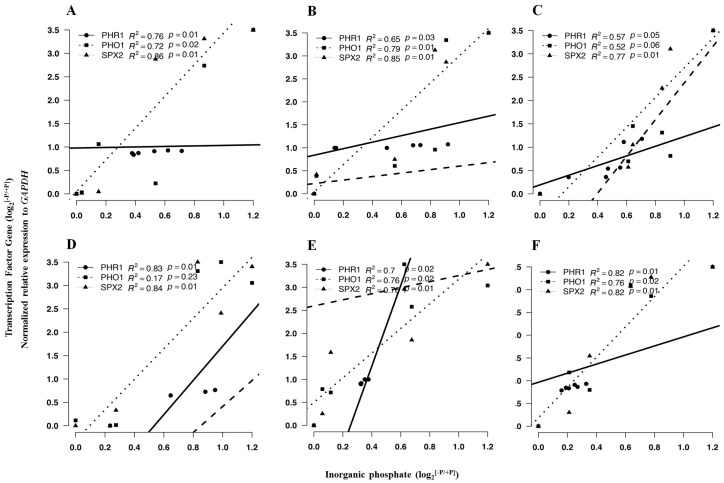
Linear correlations between the fold changes (log_2_^[−P/+P]^) of *PHO1*, *PHR1* and *SPX2*. The expression of Pi concentration in young shoots (**A**,**D**), leaves (**B**,**E**) and root (**C**,**F**) of cultivars Fengqing (**A**–**C**) and Longjing-43 (**D**–**F**).

Table 4 shows the fold changes in expression of transcription factors of *PHR1*, *PHO1* and *SPX2*. We found that all these genes were upregulated in both cultivars. The upregulation was within the comparable ranges among organs (young shoots, leaves, and root). However, their expression was more upregulated in the young shoots than in leaves and root. There were significant linear correlations between the log${}_{2}$ fold change ratio (−P/+P) of *PHR1*, *PHO1* and *SPX2* with those of Pi concentrations in most cases (Figure 4). It appeared that the transcription factor *SPX2* was more dependent on Pi in comparison to *PHR1* and *PHO1*.

#### 2.5.2. Expression of Selected Genes in Response to P Limitation

The expression of most genes investigated was upregulated in all organs of two cultivars though in some cases not statistically significant (Table 5). *Amino acid transferase 1* (*BCAT1*, EC 2.6.1.42) catalyses the last step, Val and Leu synthesis from Pyr (Figure 1). *BCAT1* expression was upregulated by P starvation in all plant organs of Fengqing (>0.85-fold) and Longjing-43 (>0.85-fold). Biosynthesis of Phe from arogenate route in plants is primarily catalyst by *arogenate dehydratase 1* (*ADT1*, EC 4.2.1.51/4.2.1.91). There was a significant upregulation effect on *ADT1* by −P treatment in all organs of both cultivars except in Longjing-43 root. Met biosynthesis was catalyzed by *methyltransferase 2* (*MS2*, EC 2.1.1.14) from homocysteine. *MS2* expression was upregulated in young shoots of both cultivar and root of Fengqing as a result of P starvation. Likewise, NADH-GOGAT (*GLT1*) was upregulated in leaves of all cultivars. The exchange between 2-oxoglutarate and 2-oxoglutamate was catalyzed by *ω-amidase, chloroplast* (*NPL3*, EC 3.5.1.111). Remarkably, the expression of *NPL3* was significantly upregulated in root and young shoots. The biosynthesis of hydroxyproline from Pro is primarily catalyst by *prolyl 4-hydroxylase* (*P4H*, EC 1.14.11.2). The expression of *P4H* was significantly upregulated by P starvation in leaves and young shoots.

The final two steps of flavonoid biosynthesis resulting in the formation of EGC and EC were catalyzed by *anthocyanidin reductase* (*ANR*, EC 1.3.1.77) (Figure 2). *ANR* expression in young shoots was not affected but was significantly upregulated in roots of both cultivars (Table 5). *Leucoanthocyanidin dioxygenase* (*LDOX*, EC 1.4.11.19) which synthesizes leucocyanidin from cyanidin was upregulated in young shoots in Longjing-43 cultivar only. During flavone and flavanol biosynthesis, glycosylation occurs in O-3 or O-7 position, which is assumed to be catalyzed by *UGT78D* family (*UGT78D1*; EC 2.4.1.159, *UGT78D2*; EC 2.4.1.91) and *UGT57L12*, EC 2.1.4.81), respectively. The expression of these *UGT* genes was upregulated by P starvation.

### 2.6. Correlation and Pathway Impact Analysis of Metabolites and Gene Expression with High-Fold Response Ratios

**Figure 5:**
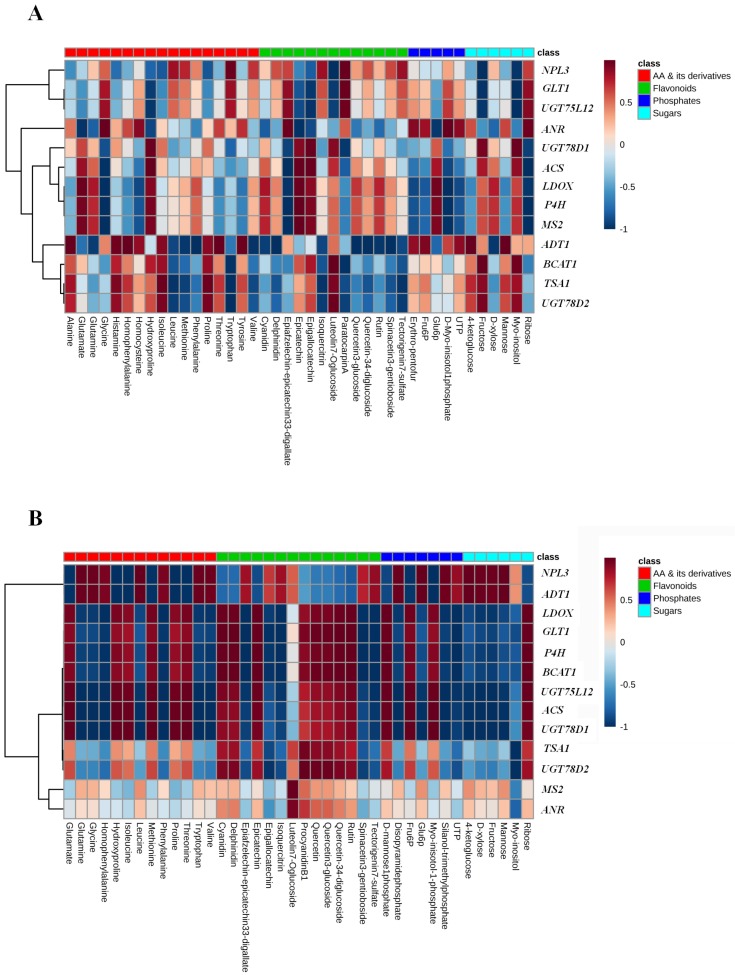
Heat map of correlations between fold change (log_2_^[−P/+P]^) of selected significantly changed (p< 0.05, *t*-test) metabolites and metabolic genes in young shoots. Red and blue colors indicate positive and negative coefficients and their scales indicate values of Fengqing (**A**) and Longjing-43 (**B**).

In the map of Fengqing (Figure 5A), we found genes were distinctly clustered into first (*NPL3*, *GLT1* and *UGT57L12*), second (*UGT78D1*, *ACS*, *LDOX*, *P4H* and *MS2*) and third (*ANR*, *ADT1*, *BCAT1*, *TSA1* and *UGT78D2*) major hierarchal groups to show their similar expression of correlations with abundance of metabolites. The significant results from first group indicate that *NPL3* positively correlated with paratocarpin A while negatively with luteolin 7-O glucoside and fructose. Similarly, *GLT1* shared strong and negative correlation with myo-inositol. Most of the gene (*P4H*, *MS2* and *ACS*) in the second group were strongly correlated with catechins. The result showed that epicatechin positively and epiafzelechin-epicatechin 3,3${}^{\prime }$-digallate negatively correlated with those genes. Glycine negatively correlated with *P4H*, *MS2* and *LDOX*. Most of the metabolites in the third group correlated with *ADT1*, either positively for Thr and Ala or negatively for Phe, delphinidin, and quercetin-3,4${}^{\prime }$-diglucoside. Likewise, in Longjing-43 (Figure 5B), *MS2* and *ANR* were distinctly clustered to an analogous pattern of correlation expression with metabolites. *MS2* strongly correlated with luteolin 7-*O* glucoside. *BCAT1*, *UGT57L12*, *ACS*, *UGT78D1*, *TSA1* and *UGT78D2* were clustered into another group *BCAT1* strongly correlated with most of the flavonoid compounds, either positively with rutin, paratocarpin A, cyanidin, delphinidin, quercetin-3,4${}^{\prime }$-diglucoside or negatively with Spinacetin 3-gentioboside and Tectorigenin 7-sulfate. *ACS*, *UGT57L12* and *UGT78D1* share the parallel negative correlation with Gln, glycine and d-xylose. Interestingly, *TSA1* share only the negative correlation with myo-inositol.

## 3. Discussion

Exposure of plants to Pi starvation has effects at morphological [27], physiological [28], biochemical [4,29,30,31,32] and molecular [33,34] levels. In this study, Longing-43 was found more sensitive to Pi-depletion than Fengqing cultivars showing different efficiency of cultivars to P use (Table 1). Starvation of P supply led to significantly decreased P concentrations in tea plants, inducing strong root morphological changes (Table 1 and Table 2). Root volume and surface area were strongly reduced whereas the lateral root length was significantly extended. This observation is in line with previous findings, as recently reviewed, that elongation of lateral root increased by P limitation [35]. However, we observed a decreased number of root tips in contrast to enhanced lateral root formation, which might be explained by the fact that response of root morphological properties is variable upon plant species and even cultivars [36]. This explanation is supported by recent findings that change in total root length, number of root tips and root surface varied significantly among barley (*Hordeum vulgare*), canola (*Brassica napus*), and potato (*Solanum tuberosum*) [37]. In previous studies, sugar (particularly sucrose) has been proposed to act as a trigger for the transcription of Pi-stress induction and Glc as transcription signal call for gene involved in cell division and respiration [38,39]. Such an effect also has been attributed to remobilization of sugars from leaves to root, causing changes in root growth parameters by P starvation. Thus, the positive changes of sugar metabolites (Supplementary Table S1; tremendously increased Fru and Glc in leaves, and Glc, d-xylose and gluconate in root) might have contributed to the changes in root parameters (Table 2).

There are several metabolomics studies and some of them integrated with metabolic genes on the response to Pi-stress [10,11,14,40]. Our analysis revealed that transcription factors *SPX2* had close relation with effect on the distribution of Pi in different plant organs that ultimately influenced on metabolic pathways. Several studies have shown that leaf and root could act as a source to provide metabolites to young shoots as sinks. For example, theanine synthesis occurs in roots and is transported above-ground and mainly young shoots [41,42]. Mature leaves are the source of carbohydrate, providing metabolites to young shoots and the metabolism of lipids [43]. We observed largely variable response of metabolites to P starvation in different organs; i.e., leaves, roots, and young shoots. Otherwise, P is highly mobile within plants and preferentially transported to the growth center young shoots, which is a tolerance mechanism of tea plants to low P supply [26]. Therefore, we conducted studies not only in young shoots but also in leaf and root.

One of the generally uniform findings under P starvation is the depletion of phosphorylated metabolites [10,14]. This was confirmed by the present work that D-mannose-1-phosphate in leaves immensely decreased (Figure 1 and Table S1). Ding et al. [24] also reported reduced contents of phosphorylated metabolites in P-deficient tea leaves. On the other hand, it was frequently observed that carbohydrates and amino acids accumulated under P starvation [10,11,14,40]. This observation is in accordance with the present result showing that carbohydrates (Fru and Glc) in leaves and some amino acids (Thr, hydroxyproline and Met) in leaves and (Phe, Ala, Trp and Tyr) in root accumulated by extremely high folds in P-starved tea plants (Table S1). Elevated carbohydrates in source leaves might be a result of weak sink requirement and use imposed by stunned growth of young shoots or/and Pi saving mechanism in which the retention of sugars in the vacuole sequesters them from phosphorylation reactions [6]. Our data, however, contradicts recent observations that Fru and Glc decreased in P-stressed tea leaves [24]. We observed opposite changes of Pyr and Ala in leaves and root (Table S1). The ratio of Pyr/Ala has been taken as an indicator of metabolic flux of Pyr to Ala synthesis or to Krebs cycle [44]. In this context, the ratio of Pyr/Ala significantly decreased in root but increased in leaves, suggesting different adjustment of N and C metabolism in leaves and root in response to P deficiency (Table S1). The accumulation of hydroxyproline under P starvation was accompanied by upregulated expression of *P4H*. The formation of 4-hydroxyproline from Pro is involved in plant responses to abiotic stress (hypoxic, anoxic, waterlogging and wounding) [45]. Our work suggests that hydroxylation of Pro by *P4H* might also be linked to the response to P deficiency. One surprising finding was that EA significantly depleted by 4.6–27.0-fold in leaves under P starvation (Table S1). A recent work demonstrated phosphoethanolamine hydrolysis catalyzed by *AtPECP1*, resulting increased EA during Pi starvation [46]. The reason for such difference is unknown and therefore requires further work. To our surprise, there was no significant change of organic acids such as citrate, malate, and oxalate in root, which were frequently reported to be exudated to rhizosphere to mine soil phosphate [25]. However, other organic acids, i.e., quinic acid and shikimic acid in root were significantly elevated. Shikimate is the precursor for Phe biosynthesis and thereby downstream metabolites of flavonoids. Increased shikimate in the root of P deficiency is consistent with elevated accumulation of flavonoids (Figure 2).

Several flavonoids accumulated in leaves (Delphinidin, cyanidin, procyanidin B1) and roots [procyanidin B1, epiafzelechin-epicatechin 3,3${}^{\prime }$-digallate, (−)-epigallocatechin 3,3${}^{\prime }$-di-gallate, malvidin and (−)-Epiafzelechin 3-gallate] of a tea plant exposed to P limitation (Figure 2 and Table S2). This agrees with previous findings [10]. Consistent with these changes, the expression of genes *ANR* and *LDOX* related to the biosynthesis pathway of these metabolites was largely upregulated. On the other hand, flavonoid glycosides in leaves (luteolin-7-*O*-glucoside, rutin, Isoquercitrin and licorice glycoside A) and roots (Diosmetin 7-*O*-beta-d-glucuronopyranoside, tricin 7-glucuronide and prunin 6${}^{\prime \prime }$-p-coumarate) were also remarkably elevated by P starvation, which is in accordance with enhanced expression of *UGT*s (*UGT78D1*, *UGT78D2* and *UGT57L12*). Flavonoids in plants are glycosylated to modified stability, solubility, localization, and thereby the biological properties. A compromised 3-*O*-glycosylation led to the repression of flavanol biosynthesis along with the inhibition of flavanol biosynthetic genes [47]. The 3-*O*-glycosylation of flavanols (kaempferol, quercetin) is catalyzed by the *UGT78D* family, with *UGT78D1* using UDP-rhamnose and *UGT78D2* using UDP-glucose [48]. The recently identified gene-encoding flavonoid 7-*O*-glycosyltransferase (*CsUGT75L12*) in tea plant displays glycosyltransferase activity on the 7-OH position of multiple phenolic compounds [49]. In the present work, we observed low levels of m-trigallic acid and 2,4-dihydroxybenzoic acid, a finding in agreement with previous ones of decreased benzoids in P-starved *Arabidopsis* [10]. Furthermore, chalcone also decreased while most flavonoids increased in young shoots under P deficiency. Competition for substrates between upstream and downstream branches of the phenylpropanoid pathway in tea plants has been suggested [50].

In young shoots, the highly accumulated carbohydrates were d-xylose and ribose, mannose and 4-ketoglucose in strongly contrast with highly increased Fru and Glc in leaves (Figure 1 and Table S1). Furthermore, d-myo-inositol 1 phosphate remarkably increased whereas myo-inositol decreased. Inositol and its derivatives are crucial for development and signaling in plants, either as metabolic mediators or participating in various signaling pathways in response to stress, hormones, and nutrients, by transcriptional regulation of the stimuli-responsive genes [51]. Declining myo-inositol biosynthesis in leaves led to programmed cell death in *Arabidopsis mips1* mutants [52,53]. On the other hand, we observed increased phosphorylated metabolites (d-myo-inositol 1 phosphate and d-erythro-Pentofuranose phosphate) in young shoots in contrast to those in leaves and roots (Figure 1 and Table S1). This might be explained by the fact that these metabolites play important roles and are therefore essentially maintained at higher levels for new growth under P limitation. Both metabolomics and targeted analyses showed remarkably lower level of Gly in young shoots of both cultivars exposed to −P treatment (Table S1 and Table 3). Though Ser contents responded different between the two cultivars, the ratio Gly/Ser was greatly decreased by −P treatment (5.0, 3.9 at P sufficiency and 1.8, 0.6 at P deficiency for Fengqing and Longjing-43, respectively) (Table 3). The ratio Gly/Ser is a good marker of photorespiration and correlates well with the flux of C through the photorespiratory pathway, regardless of the absolute amounts of each amino acid [54]. Significantly decreased Asp concentrations in both cultivars further supported the conclusion that photorespiration in young shoots was repressed by P deficiency [55]. This finding, however, is in contrast with increased Gly/Ser ratio after short-term P deficiency and activated photorespiration by P stress [6,56]. Increase in amino acid Pro in our study corresponds to previous one in the tea plant [23], indicating that P stress accelerates Pro accumulation. A recent work in *Arabidopsis* showed the link of Pro metabolism and P nutrition and revealed that the activated Pro biosynthesis is regulated by cross talk between ABA signaling and regulation of phosphate homeostasis through *PHR1* [57]. Therefore, depleted myo-inositol, abnormal Pro accumulation and inactivated photorespiration likely mean the strongly stunted growth of young shoots due to P limitation.

The quality of tea is dependent upon chemical components in young shoots. Therefore, the targeted metabolites in young shoots were further determined. Earlier investigation showed that P-deficient green tea displayed decreased concentrations of total free amino acid, total polyphenol and individual components Thea, Asp, Glu and ECG, EC, GCG and CG without affecting total catechins and EGCG [23]. The present result suggested, however, a more complicated response of these metabolites to P stress, especially between the two cultivars. The uniformly changed metabolites within the two cultivars include amino acids Asp, Gly, Leu, Pro, Ile, and catechin. Other amino acids including the predominating Thea and others (Ser, Thr, Glu, Val, Met, Phe), and some catechins (EGC and ECG) displayed opposite changes in response to P stress between the two cultivars.

## 4. Materials and Methods

### 4.1. Plant Cultivation, P Treatment and Sampling

The six-months-old seedlings of the cultivar Longjing-43 (12.0 ± 0.4 g) (Tea Research Institute, Chinese Academy of Agricultural Sciences (TRI CAAS), Hangzhou, Zhejiang, China) propagated from rooted-cuttings and Fengqing (5.9 ± 0.3 g) (Tea Research Institute, Fengqing, Yunnan, China) germinated from seeds were cultivated in pots filled with 4 L nutrient solutions. The basic composition of nutrient solutions were macronutrients(mM) (NH${}_{4}$)${}_{2}$SO${}_{4}$(2), Ca(NO${}_{3}$)${}_{2}$(1), K${}_{2}$SO${}_{4}$(1), MgSO${}_{4}$(0.4), CaCl${}_{2}$(0.8) and micronutrients(μM) FeEDTA(0.08), H${}_{3}$BO${}_{3}$(10), MnSO${}_{4}$(1.5), ZnSO${}_{4}$, CuSO${}_{4}$(0.2) and (NH${}_{4}$)${}_{6}$Mo${}_{7}$O${}_{2}4$(0.5) (Nutrition Chemicals; Shanghai Lingfeng Chemical Reagent Co. Ltd., Shanghai, China). The level of P was 0.1 mM solution supplied as KH${}_{2}$PO${}_{4}$, whereas rest of the seedlings grown without P. The pH (pH meter; Orion A215, Thermo Fisher Scientific Inc., Waltham, MA, USA) of nutrient solutions was daily adjusted to 5.0 by adding 0.1 M H${}_{2}$SO${}_{4}$ or NaOH solution. There were 8 pot replications or 32 plants for each cultivar (*cv*.). The plants were cultivated for three months in a greenhouse under natural light 25,643 ± 3987 LUX (LiCor LI-250A connected to quantum sensor (LI-190SA), Lincoln, NE, USA) at room temperature (25 ± 2 ${}^{\circ }$C). During this period, young shoots were sampled 4–6 times after 90 days of exposition to P treatments from the plants whenever they reached the desired developmental stage for harvesting, which is the common standard for green and black tea. In addition, leaf and fibrous root were also sampled as they act as source provide metabolites to young shoots as sink. All plant samples including young shoots, leaves and roots were quickly frozen in liquid nitrogen and stored in an ultra-low temperature refrigerator at −80 ${}^{\circ }$C for further analysis.

### 4.2. Measurements of Root Growth Parameters and Concentrations of Pi

A weighted batch of fresh fibrous root sample was randomly selected from the collected samples and was scanned with an Epson V700 scanner (B11B178011, EPSON, Long Beach, CA, USA) in 600 dpi. Parameters of root volume (cm${}^{3}$), surface area (cm${}^{2}$), total number of tips, length (cm) and thickness (cm) were calculated from the images using Root Graph phenotyping program, by setting the threshold scalar at 0.6 to remove noise [58]. Pi concentration in freeze-dried plant samples was measured by ICP-AES (iCAP6300DUO, Thermo Fisher Scientific Inc., Waltham, MA, USA) following digestion with concentrated HNO${}_{3}$ and HClO${}_{4}$ [59].

### 4.3. GC×GC-TOF/MS Analysis for Primary Metabolites

Freeze-dried young shoots, leaves, and root samples (100.00 mg) with four biological replicates each was added to 1000 μL of methanol-chloroform (3:1, *v*/*v*) solvent (Sigma-Aldrich Co., St. Louis, MO, USA). 10 μL L-2-chlorophenylalanine (0.3 mg/mL in water) was mixed with the resultant extract. The samples were centrifuged for 10 min at 12,000 rpm at −4 ${}^{\circ }$C; the supernatant (400 μL) was dried in a vacuum concentrator without heating, and later frozen in liquid nitrogen. 80 μL methoxyamine (15 mg/mL in pyridine) was added to the dried sample and vortexed for 1 min. Methoxymation was performed at 37 ${}^{\circ }$C for 120 min in drying oven. Subsequently, the sample were trimethylsilylated at 70 ${}^{\circ }$C for 90 min by adding 80 μL BSTFA (containing 1% TMCS) to the solution.

The machine consisted of an Agilent GC 6890N gas chromatograph, the high-speed TOF mass spectrometer detector (Pegasus HT, Leco Co., St. Joseph, MI, USA). First dimensional, DB-5 MS capillary column (30 m × 250 μm i.d., 0.25 μm film thickness; JandW Scientific, Folsom, CA, USA) and second dimension column DB-17H 2.5 m × 0.1 mm I.D., 0.1 μm film thickness. The rest of the machine conditions were according to the previously described in method by Liu et al. [60].

Data files from GC×GC-TOF/MS were processed in Leco software (Leco Co., St. Joseph, MI, USA) and were deconvoluted using System. The corresponding peaks of chromatogram were compared to the NIST mass spectral database (National Institute of Standards and Technology, FairCom Co., Gaithersburg, MD, USA) to identify metabolites.

### 4.4. UPLC-Q-TOF/MS Analysis for Secondary Metabolites

Samples stored at −80 ${}^{\circ }$C in liquid nitrogen were finely milled. The milled four biological replicated samples were weighed to exactly 0.1 g with the help of ice crucible in Eppendorf tube. The metabolites in weighed samples were extracted using 80% methanol and 1% formic acid (Sigma-Aldrich Co., St. Louis, MO, USA) as indicated by [50,61]. The extracted 2 mL solvent were bathed ultrasonically for 10 min, while turning the hands both up and down twice in 5 min interval, and after centrifuged at 12,000 r/min. Extracts were filtered through a 0.22 μm PTFE filters before injection into 1 dram glass vessel for metabolomics analysis.

Metabolomics analysis was performed on an ultra-performance liquid chromatography (ACQUITY UPLC, Waters Corp., Milford, MA, USA), which was equipped with an Acquity HSS T 3 columns (1.8 mm, 100 mm 62.1 mm, Waters Corp., Milford, MA, USA) and connected to a quadrupole-time of flight mass spectrometer (Xevo G2-XS QTOF, Waters Corp., Milford, MA, USA). Mobile solutions and rest of the machine conditions were adjusted as previously referred to in method [50].

Raw chromatographic data acquired from the UPLC-Q-TOF/MS analysis were exported into a comma delimited file (*.csv) by TransOmics (Waters Corp., Milford, MA, USA). Metabolite peaks were annotated from the accurate mass measurements using online metabolite databases. Peaks were identified based on (i) actual mass (AM), retention time (RT) and standard; (ii) AM and RT; (iii) AM and isotopic distribution (ID) as prescribed by [50]. The identified peaks can be further classified into identified compounds (i and ii) and putatively annotated compounds (iii) according to the proposed minimum reporting standards for chemical analysis [62].

### 4.5. Extraction and Determination of Targeted Catechin and Amino Acid by High Performance Liquid Chromatography (HPLC) Analysis

Samples were assigned and extracted same as UPLC-Q-TOF/MS for catechin determination. For free amino acid analysis samples were derivatized as the instruction provided by waters AccQ.Tag chemistry package. HPLC analysis was carried out using an e2695 connecting 2998 photodiode array detector system (Waters) injected with 10 μL and 25 μL of sample solutions for catechin and free amino acid respectively. For catechins distilled water with 2% formic acid was used as mobile phase A. Mobile phase B consisted of HPLC solvent ACN (Sigma-Aldrich Co., St. Louis, MO, USA). The samples were eluted at column temperature 40 ± 1 ${}^{\circ }$C at a flow rate of 1 mL/min and monitored at 278 nm. Similarly, for amino acid AccQ.Tag eluent from waters was used as mobile phase A. Mobile phase B was ACN and column temperature was set at 37 ± 2 ${}^{\circ }$C. Rests of the procedure was followed as prescribed in AccQ. Tag chemistry package instruction manual. Peaks of catechins were identified by comparing the retention time of the sample to those of authentic standards and amino acids were as prescribed in the manual.

### 4.6. Quantitative Real-Time Polymerase Chain Reaction (QRT-PCR) Expression Analysis

Young shoots and leaves from Pi-starved (−P) and Pi-sufficient plants (+P) of two cultivars were used for RNA extraction. RNA was extracted from fresh young shoots, leaves of P-starved and P-sufficient plants each in three biological replicates using the RNeasy Plant Mini kit following the protocol provided by the producer [63] and from root following prescription by Tao et al. [64].

cDNA synthesis using PrimeScript RT Reagent Kit according to manufacturer instructions was carried out. cDNA quality from young shoots, leaves, and root from +P and −P were tested. The transcript of three Pi transporter genes and expression of 13 phosphate starvation selected metabolic genes was quantified using a 7500 HT Real-Time PCR system (Applied Biosystems, Foster, CA, USA). The three-transcription factor gene (*PHR1*, *PHO1* and *SPX2*) and 13 metabolic genes were analyzed as fold change after normalizing C_T_ with the reference gene *GAPDH* [65]. In addition, three-transcription factor use linear regression with respect to Pi content change in different plant organs to formulate the dependent factor, whereas the rest of the genes were shown as fold difference in expression. The primer sequences of the gene used for qRT-PCR are shown in Tables S3–S5.

To integrate primary and secondary metabolites changes with gene expression, the fold changes of primary and secondary metabolites were checked with the corresponding expression of pathway metabolic genes. qRT-PCR expression profiling involving 13 genes transcript were performed in leaves, root, and young shoots of Fengqing and Longjing-43 cultivar. The gene was selected from in between the pathway metabolites that were derived as VIP from SIMCA software and were further referenced from Kyoto Encyclopedia of Genes and Genomes (KEGG) database (http://www.genome.jp/kegg/) and Plant Metabolic Network (PMN) (www.plantcyc.org).

### 4.7. Data Analysis, Visualization, and Cross Verification

Pi content in root, leaves and, young shoots and targeted metabolites amino acid and catechins content in young shoots measurements were entered into MS Excel 2013 (Microsoft Co., Redmond, WA, USA), and later an analysis of variance test and Pearson’s correlation on selected untargeted quality-related metabolites was performed at 95% confidence interval (CI) or 0.05 level of significance in SPSS (Version 22, IBM, Chicago, IL, USA.) software. Figures were prepared using Power Point 2013 (Microsoft Co., Redmond, WA, USA), HeatMapper Plus Tool (http://bar.untoronto.ca) or Cytoscape 3.02 (http:// www.cytoscape.org/) and graphs using R 3.2.3. SIMCA 13.0.3 (Umetrics, MKS Instruments Inc., Umeå, Sweden) software was used to apply umetrics model to sort GC×GC-TOF/MS and UPLC-Q-TOF/MS data.

The UPLC-Q-TOF/MS and GC×GC-TOF/MS data were exported to SIMCA 13.0.3 software for multivariate data analyses. An unsupervised principal component analysis (PCA) was performed for the data obtained from GC×GC-TOF/MS and UPLC-Q-TOF/MS for a general overview of the variance of metabolites in the treatments using SIMCA-P (version 13.0, Umetrics, MKS Instruments Inc., Umeå, Sweden). The supervised orthogonal projection to latent structure discriminant analysis (OPLS-DA) was carried out to obtain cluster information on differences in the treatment. The VIP values of all the data from the double cross and 7-fold cross validated OPLS model, was taken as coefficients for metabolite selection for GC×GC-TOF/MS and UPLC-Q-TOF/MS, respectively. Those variables with VIP > 1 were selected and a student *t*-test was performed on a different group of plant organs for treated metabolites, to test for significance of the selected group of metabolites, as well as on each metabolite. Response ratio (−P/+P) of significantly changed parameters of root growth and P concentrations and metabolites was log${}_{2}$ transformed and referred to as ‘fold change’ to show the effects of −P treatment. The heatmap of correlation matrices was built to show the correlation relation between metabolites and metabolic gene in young shoots.

## 5. Conclusions

In summary, P deficits increase in carbohydrates (fructose and glucose) and amino acid metabolites such as threonine and methionine cause weak sink requirement with low production rate. The different amount of higher and upregulated change response of Pi in roots and interlinkage of this change with *SPX2* in other organs further explains Longing-43 is more sensitive to Pi-depletion than Fengqing cultivar. Some amino acid metabolites, such as phenylalanine, alanine, tryptophan, and tyrosine accumulated by extremely high fold in root during this occasion. The predominating theanine and other amino acids (serine, threonine, glutamate, valine, methionine, phenylalanine), and catechin (EGC, EGCG and CG) content explains two tea cultivars have a different metabolic response to Pi deficiency. The consistent changes in numbers of amino acids and flavonoids in biosynthetic pathway with expression profile of genes (*ANR*, *LDOX* and *UGT*s) revealed cultivars leading molecular regulatory network mechanisms behind P deficiency.

## Figures and Tables

**Table 1:** Concentrations of phosphorus (mg·g${}^{-1}$) in plants supplied with two P levels in nutrient solutions.

Cultivar	P Level	Young Shoots	Leaves	Root	Whole Plant
Fengqing	−P	2.12 ± 0.5b	2.39 ± 1.31b	2.87 ± 1.41b	2.46 ± 1.04b
Fengqing	+P	3.80 ± 0.8a	3.94 ± 0.83a	7.17 ± 2.66a	4.97 ± 1.41a
Longjing-43	−P	2.19 ± 1.5b	3.81 ± 1.00b	2.15 ± 0.57b	2.72 ± 0.93b
Longjing-43	+P	12.05 ± 5.63a	7.15 ± 2.16a	9.15 ± 2.44a	9.45 ± 3.33a

a and b within the column of the same cultivar indicate significant differences between P treatments (p< 0.05).

**Table 2:** Fold change of response to P treatments (log_2_^[−P/+P]^) in mean volume, surface area, length, average thickness, and tips of roots.

Parameter	Fengqing	Longjing-43
Volume	−1.54 *	−2.84 *
Surface area	−1.67 *	−1.99 *
Number of root tips	−1.96 *	−0.77 *
Length	2.32 *	0.49 *
Average thickness	0.15 *	0.01^NS^

NS and * indicates insignificant or significant (p< 0.05) differences between the two P levels.

**Table 3:** Targeted metabolites amino acid and catechins (mg·g-1) in young shoots of tea plants supplied with two P levels in nutrient solutions.

Metabolites	Fengqing		Longjing-43	
	−P	+P	−P	+P
*Amino acids*				
Asp	2.06 ± 0.16b	3.13 ± 0.05a	1.36 ± 0.05b	2.47 ± 0.22a
Gly	0.29 ± 0.01b	1.55 ± 0.14a	0.11 ± 0.01b	0.47 ± 0.04a
Leu	0.28 ± 0.01b	0.45 ± 0.04a	0.11 ± 0.01b	0.20 ± 0.01a
Pro	3.56 ± 0.13a	1.95 ± 0.04b	1.66 ± 0.13a	0.84 ± 0.01b
Ile	1.21 ± 0.05a	0.86 ± 0.08b	0.43 ± 0.02a	0.22 ± 0.02b
Glu	9.64 ± 0.77a	7.78 ± 0.13b	2.36 ± 0.19b	3.41 ± 0.06a
Val	0.42 ± 0.01a	0.55 ± 0.05b	0.46 ± 0.02b	0.62 ± 0.06a
Met	2.52 ± 0.10a	1.97 ± 0.78b	1.08 ± 0.04b	1.44 ± 0.3a
Phe	1.19 ± 0.05a	0.93 ± 0.01b	0.52 ± 0.02b	1.18 ± 0.2a
Thea	8.52 ± 0.68b	20.81 ± 0.36a	22.78 ± 1.83a	17.02 ± 0.29b
Ser	0.16 ± 0.02b	0.31 ± 0.03a	0.20 ± 0.02a	0.12 ± 0b
Thr	0.24 ± 0.01b	0.38 ± 0.34a	0.78 ± 0.03a	0.55 ± 0.05b
Arg	1.18 ± 0.05a	0.78 ± 0.01b	0.41 ± 0.35 ^NS^	0.24 ± 0.25
Tyr	0.21 ± 0.15 ^NS^	0.43 ± 0.01	0.45 ± 0.05a	0.24 ± 0.03b
His	0.59 ± 0.17 ^NS^	0.52 ± 0.13	0.55 ± 0.14 ^NS^	0.53 ± 0.18
Ala	0.11 ± 0.02 ^NS^	0.08 ± 0.01	0.08 ± 0.04 ^NS^	0.11 ± 0.03
Cys	0.40 ± 0.25 ^NS^	0.49 ± 0.36	0.29 ± 0.08 ^NS^	0.56 ± 0.37
Lys	0.81 ± 0.49 ^NS^	1.72 ± 1.02	0.61 ± 0.39 ^NS^	1.17 ± 0.51
*Catechins*				
EGCG	34.03 ± 1.36a	25.77 ± 2.34b	33.25 ± 6.5 ^NS^	36.44 ± 0.4
EGC	6.92 ± 0.56a	4.25 ± 0.07b	3.02 ± 0.24b	10.93 ± 0.19a
ECG	2.71 ± 0.22a	2.24 ± 0.04b	2.80 ± 0.22b	3.40 ± 0.06a
GC	0.14 ± 0.05a	0.03 ± 0.02b	0.05 ± 0 ^NS^	0.07 ± 0.03
CG	0.03 ± 0b	0.05 ± 0.01a	0.06 ± 0.01a	0.02 ± 0b
C	1.06 ± 0.04a	0.66 ± 0.06b	1.37 ± 0.04a	0.96 ± 0.19b
GCG	0.19 ± 0.04 ^NS^	0.20 ± 0.02	0.23 ± 0.01 ^NS^	0.16 ± 0.03

Means with different letter and NS indicates significant or not significant (p< 0.05) differences between P treatments.

**Table 4:** qRT-PCR expression of transcription factor genes normalize with reference gene delta-CT (ΔC_T_) are shown as fold changes (log_2_^[−P/+P]^).

Genes	Fengqing			Longjing-43		
	Young Shoots	Leaves	Root	Young Shoots	Leaves	Root
*PHR1*	0.89 ***	1.03 *	0.69 *	0.01 *	0.95 *	0.86 **
*PHO1*	1.01 *	1.21 ^NS^	0.70 *	0.81 ^NS^	0.78 *	1.20 ***
*SPX2*	1.70 **	1.21 *	1.53 ***	1.76 ***	0.82 ***	0.55 ***

NS, ***, ** and *: indicate not significant or significance at 0.001, 0.01, and 0.05 levels between P treatments by *t*-test, respectively.

**Table 5:** qRT-PCR expression of metabolic gene transcripts normalized with reference gene delta-CT (ΔC_T_) are shown as fold changes (log_2_^[−P/+P]^).

Genes	Fengqing			Longjing-43		
	Young Shoots	Leaves	Root	Young Shoots	Leaves	Root
*BCAT1*	0.88 **	1.15 *	0.86 **	0.88 **	0.85 *	0.92 **
*TSA1*	1.19 *	1.50 **	1.12 ***	1.61 ***	0.85 ***	0.92 ***
*ADT1*	1.55 ***	1.09 *	0.87 *	0.91 **	1.15 **	0.67 ^NS^
*MS2*	0.92 **	1.03 ^NS^	0.86 *	1.67 ***	0.98 ^NS^	0.99 ^NS^
*NPL3*	0.98 *	0.93 ^NS^	0.85 **	0.58 ***	1.05 *	0.92 *
*GLT1*	0.88 *	1.56 **	0.63 **	0.71 **	0.90 *	1.01 ^NS^
*ACS*	0.85 **	1.34 **	0.87 ***	1.62 ***	0.92 *	1.19 ***
*P4H*	0.96 *	1.30 **	1.01 ^NS^	1.62 **	0.91 *	1.27 *
*ANR*	1.02 ^NS^	2.08 ^NS^	1.06 *	1.16 ^NS^	0.63 **	0.56 ***
*LDOX*	0.96 ^NS^	0.95 *	1.10 ^NS^	1.74 **	0.62 ***	1.02 ^NS^
*UGT75L12*	0.83 **	1.04 *	0.80 **	2.17 ***	0.89 **	1.17 *
*UGT78D1*	0.92 *	1.46 *	0.96 *	1.19 *	0.92 *	0.97 ^NS^
*UGT78D2*	0.84 ***	1.17 *	0.70 ***	1.70 ***	0.86 **	0.81 *

NS, ***, ** and *: indicate not significant or significance at 0.001, 0.01, and 0.05 levels between P treatments by *t*-test, respectively.

## Supplementary Materials

The following are available online at http://www.mdpi.com/1422-0067/19/11/3683/s1, Figure S1: Supervised and Unsupervised OPLS-DA and Permutations test, with variables phosphate starvation (−P) and phosphate sufficient (+P) samples, Table S1: Fold changes (log_2_^[−P/+P]^) and *t*-test of selected metabolites in tea plants in response to P limitation measured by GC×GC-TOF/MS, Table S2: Fold changes (log_2_^[−P/+P]^) and *t*-test of selected metabolites in tea plants in response to P limitation measured by UPLC-Q-TOF/MS. Table S3: Transcription factor primer sequences for Fengqing (*cv*. *assamica*) and Longjing-43 (*cv*. *sinensis*) for qRT-PCR analysis., Table S4: Primer sequences for the gene transcripts of Fengqing (*cv*. *sinensis*) cultivar of selected pathways for qRT-PCR analysis., Table S5: Primer sequences for the gene transcripts of Longjing-43 (*cv*. *assamica*) cultivar of selected pathways for qRT-PCR analysis.
