# Supplementary material for: Metabolic Changes of Amino Acids and Flavonoids in Tea Plants in Response to Inorganic Phosphate Limitation
Source: Int J Mol Sci. 2018 Nov 21;19(11):3683. doi: 10.3390/ijms19113683 (PMC6274676; doi:10.3390/ijms19113683)
Supplement: Supplementary file 1 [file ijms-19-03683-s001.pdf]

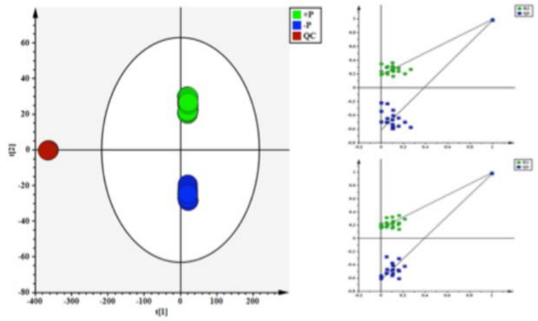

Unsupervised OPLS-DA and Permutations test of Primary Metabolites

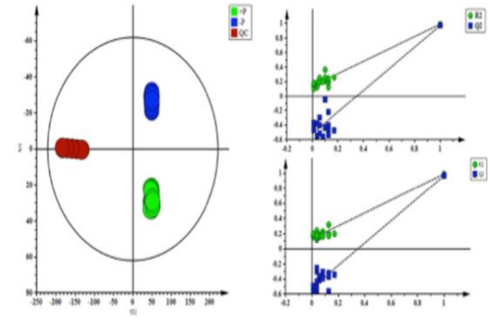

Supervised OPLS-DA and Permutations test of Primary Metabolites

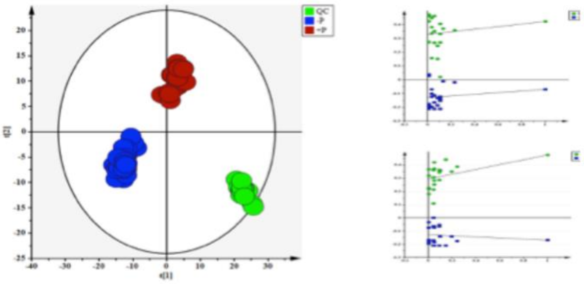

Unsupervised OPLS-DA and Permutations test of Secondary Metabolites

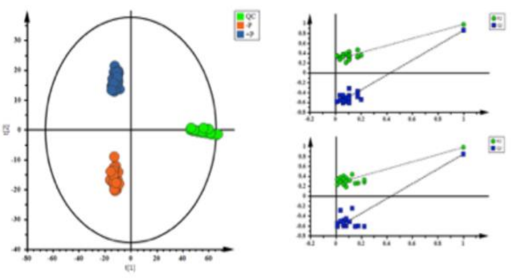

Supervised OPLS-DA and Permutations test of Secondary Metabolites

Figure S1. Supervised and unsupervised OPLS-DA and permutations test, with variables phosphate starvation (-P) and phosphate sufficient (+P) samples. Score plot and permutations sequencing tests of metabolites analysed by GC/GC-TOF/MS and UPLC-Q-TOF/MS in Fengqing and Longjing-43 cultivar. Test for Quality control (QC), -P and +P

Table S1. Fold changes ($\log_2^{[-P/+P]}$) and *t-test* of selected metabolites in tea plant organs in response to P limitation measured by GC×GC-TOF/MS

Class	Compound	Mass	1st DT (minutes)	2nd DT (seconds)	VIP	**Young Shoot $\log_2^{[-P/+P]}$**				**Leaves $\log_2^{[-P/+P]}$**				**Root $\log_2^{[-P/+P]}$**			
						Fengqing	***P-value (t-test)***	Longjing-43	***P-value (t-test)***	Fengqing	***P-value (t-test)***	Longjing-43	***P-value (t-test)***	Fengqing	***P-value (t-test)***	Longjing-43	***P-value (t-test)***
Phosphates	D-Mannose 1 phosphate	387	19.6	2.22	1.90326	1.04	<0.001	0.75	<0.001	**-29.45**	<0.01	**-26.52**	<0.001	-0.73	<0.001	-0.33	<0.001
	Fructose 6-phosphate	315	33.3	0.01	1.39642	**-30.89**	<0.01	**27.12**	<0.001	1.36	<0.001	0.15	NS	-1.41	<0.001	-0.16	<0.001
	Glucose-6-Phosphate	387	23.8	0.04	1.90992	**-6.79**	<0.001	**5.5**	<0.001	0.45	<0.05	0.65	<0.01	-0.21	<0.001	-1.91	<0.001
	D-Myo-Inositol 1 phosphate	259	33.5	1.86	1.38598	**27.36**	<0.05	**26.44**	<0.05	**-0.48**	<0.001	**-0.35**	<0.01	-1.68	<0.001	-0.56	<0.001
	D-Erythro-Pentofuranose phosphate	211	31.5	2.82	1.12797	**32.59**	<0.001	**0.15**	<0.001	-0.84	<0.05	0.26	<0.001	0.44	<0.001	0.71	<0.001
Sugars	Fructose	305	33.3	0.03	1.84388	-0.37	<0.01	-0.15	<0.05	**24.72**	<0.05	**25.6**	<0.01	0.19	<0.001	0.09	<0.001
	Glucose	287	28.8	2.32	1.75573	0.95	<0.001	0.11	NS	**24.75**	<0.001	**25.82**	<0.001	3.21	<0.001	0.98	<0.001
	D-Xylose	204	21.5	0.04	1.75318	**3.65**	<0.001	**3.51**	<0.001	-0.56	<0.01	-0.77	<0.001	1.03	<0.001	2.11	<0.001
	Gluconate	174	35	2.1	1.23656	0.23	<0.05	0.11	NS	3.07	<0.001	0.23	<0.05	**2.25**	<0.001	**3.24**	<0.001
	Ribose	50	8.4	1.4	1.61528	**27.62**	<0.001	**30.33**	<0.001	-0.01	NS	-0.76	<0.001	2.5	<0.001	A/P	-
	Mannose	205	23.2	2.35	1.37363	**3.01**	<0.001	**27.33**	<0.001	0.56	<0.001	0.13	NS	-0.45	<0.001	-1.17	<0.001
	4-Ketoglucose	232	21.7	2.92	1.24563	**27.49**	<0.001	**3.77**	<0.001	-1.01	<0.001	0.6	<0.001	3.62	<0.001	-0.54	<0.001
	Myo-Inositol	44	37.2	1.4	1.27525	**-28.46**	<0.01	**-27.53**	<0.01	1.42	<0.05	-0.07	<0.001	3.04	<0.001	-0.07	<0.001
Amino Acids & N compounds	Threonine	209	21.5	0.05	1.65724	**-26.72**	<0.05	**24.66**	<0.001	**23.1**	<0.01	**22.98**	<0.01	-0.28	<0.001	-1.49	<0.001
	Hydroxyproline	230	6.3	0.05	1.36545	-0.32	<0.01	-0.77	<0.001	**26.83**	<0.01	**23.65**	<0.01	0.32	<0.001	1.4	<0.001
	Phenylalanine	244	8.3	0.02	1.27286	0.6	<0.001	-0.63	<0.01	-2.54	<0.001	0.5	<0.001	**2.6**	<0.001	**4.63**	<0.001
	Asparagine	159	18	0.04	1.65589	-0.74	<0.05	-0.27	<0.001	P/A	-	-24.11	<0.01	-0.34	<0.001	-3.59	<0.001
	Ethanolamine	220	30.8	0.06	1.27224	0.9	<0.001	0.37	<0.001	**-4.56**	<0.001	**-27.05**	<0.001	0.49	<0.001	-0.16	<0.001
	Alanine	30	15.5	0.02	1.68114	0.44	<0.001	-0.87	<0.001	1	<0.001	0.2	<0.05	**16.89**	<0.001	**19.71**	<0.001
	Tryptophan	202	36.1	0.05	1.25853	1.08	<0.001	0.55	<0.05	0.42	<0.05	0.44	<0.001	**5.22**	<0.001	**22.85**	<0.001
	Tyrosine	180	26.5	0.05	1.12788	-2.98	<0.05	3.11	<0.001	-3.09	<0.001	0.36	<0.01	**4.41**	<0.001	**9.52**	<0.001
	Isoleucine	159	21.5	0.05	1.66455	**27.35**	<0.001	**25.13**	<0.05	-0.72	<0.001	-0.05	NS	2.8	<0.001	-0.55	<0.001
	Proline	243	6.3	0.05	1.41583	**32.75**	<0.001	**30.02**	<0.05	-0.46	<0.001	-0.87	<0.05	-0.98	<0.001	-1.94	<0.001
	Glycine	178	36.8	0.04	1.35468	**-1.84**	<0.001	**-25.43**	<0.001	1.23	<0.001	-0.41	<0.001	0.63	<0.001	-1.68	<0.001
	Leucine	95	5.7	0.06	1.59664	**-29.87**	<0.01	**-28.68**	<0.05	-0.62	<0.001	0.19	<0.01	1.96	<0.001	2.2	<0.001
	Glutamine	46	21.5	0.05	1.52591	-0.49	<0.05	1.12	<0.01	0.13	<0.001	-1.46	<0.001	-0.19	<0.001	0.98	<0.001
	Serine	73	12.6	0.03	1.14362	-0.73	<0.05	0.25	<0.01	1.23	<0.05	-0.48	NS	-0.62	<0.01	-1.71	<0.001
	Methionine	115	21.5	0.05	1.35569	0.13	<0.01	-1.46	<0.001	**5.89**	<0.01	**3.32**	<0.001	0.56	<0.01	0.65	<0.001

	Glutamate	98	8.8	2	1.32546	0.79	<0.001	-1.55	<0.001	1.07	<0.001	-0.8	<0.001	-1.08	<0.001	-2.57	<0.001
	Glutamic acid	46	17	0.04	1.23145	0.95	<0.001	-0.63	<0.05	0.56	<0.01	0.13	<0.001	1.06	<0.001	1.3	<0.001
	Glutaric acid	230	17	2.39	1.32532	0.91	<0.001	-0.06	<0.01	-0.53	<0.01	1.23	<0.01	-1.08	<0.001	-0.74	<0.001
	Lysine	46	7.6	1.75	1.09621	-0.81	<0.001	-1.62	<0.05	-0.63	<0.01	-0.38	<0.01	-1.45	<0.001	-0.23	<0.001
	Ornithine	70	21.4	2.52	1.23614	-1.41	<0.001	-2.81	<0.001	-0.47	<0.001	0.18	NS	-2.63	<0.001	3.09	<0.001
	Valine	207	10	1.8	1.22545	-0.37	<0.01	-0.76	<0.001	-0.82	<0.001	-	-	-0.12	<0.001	1.45	<0.01
Organic acid	Butanoic acid	315	18.9	0.07	1.09515	1.07	<0.001	-1.07	<0.01	**21.51**	<0.01	**26.77**	<0.05	-0.23	<0.001	-2.4	<0.001
	Pyruvate	50	7.9	1.4	1.32564	-0.08	<0.05	-0.04	<0.05	**6.64**	<0.001	**24.76**	<0.01	**-2.04**	<0.001	**-2.4**	<0.001
	Benzenebutanoic acid	98	21.5	0.05	1.33264	-0.74	<0.01	-0.94	<0.05	P/A	-	24.11	<0.01	-3.09	<0.001	1.55	<0.001
	Pentanedioic acid	158	13	2.13	1.12749	0.86	<0.001	-1.63	<0.001	**-27.9**	<0.001	**-27.73**	<0.001	0.86	<0.001	0.52	<0.001
	2-ketoglutarate	173	22.3	2.99	1.20838	**26.14**	<0.001	**27.16**	<0.001	0.47	<0.05	-0.28	<0.05	1.96	<0.001	2.11	<0.001

'NS' indicates not significant (p<0.005) differences between P treatments.

Table S2. Fold changes (log$_2$$^{[-P/+P]}$) and *t-test* of selected metabolites in tea plant organs in response to P limitation measured by UPLC-Q-TOF/MS

Class	Compound	Monoisotopic Mass	Retention Time	Adduct	VIP	Young Shoot log$_2$$^{[-P/+P]}$				Leaves log$_2$$^{[-P/+P]}$				Root log$_2$$^{[-P/+P]}$				[a]IC
						**Fengqing**	***P-value (t-test)***	**Longjing-43**	***P-value (t-test)***	**Fengqing**	***P-value (t-test)***	**Longjing-43**	***P-value (t-test)***	**Fengqing**	***P-value (t-test)***	**Longjing-43**	***P-value (t-test)***	
Flavonoids	Luteolin-7-O-glucoside	448.1005615	4.96	M-H2O-H	1.07890	**24.9**	<0.001	-	<0.001	**13.99**	<0.001	**13.08**	<0.001	**0.43**	NS	**2.06**	<0.01	(i)
	Delphinidin	337.0149	3.9	M-H	1.21593	**0.87**	<0.05	**2.25**	<0.001	**12.16**	<0.001	**1.42**	<0.001	P/A	-	P/A	-	(iii)
	Cyanidin	286.0477381	3.16	M+NA-2H/M+FA-H	1.28302	**1.81**	<0.001	**2.96**	<0.001	A/P	-	**15.07**	NS	P/A	-	P/A	-	(iii)
	Epigallocatechin	306.0739528	2.72	M+FA-H	1.42885	P/A	-	**-14.8**	<0.001	A/P	-	**21.54**	<0.001	P/A	-	A/P	-	(i)
	Rutin	610.1533849	4.5	M-H2O-H	1.56987	**3.53**	<0.001	**-1.17**	<0.001	**0.12**	<0.01	**15.73**	<0.001	16.88	<0.001	-	-	(i)
	Isoquercitrin	464.0954761	4.85	M-H	1.54177	**17.93**	<0.001	**7.04**	<0.001	**14.15**	<0.05	**0.51**	<0.01	-	-	-15.2	<0.001	(i)
	Procyanidin B1	578.1424263	2.62	M-H2O-H	1.55174	**-4**	<0.001	**-2.08**	<0.001	**9.48**	<0.001	A/P	-	14.15	<0.001	0.11	<0.05	(i)
	Kaempferol-3-O-glucoside	448.1006	5.88	M-H	1.09133	**15.07**	<0.001	**-1.93**	<0.001	0	-	-6.23	<0.001	-1.89	<0.01	-0.33	<0.05	(i)
	Quercetin-3,4'-diglucoside	324.1144	3.9	M-H2O-H	1.28735	0.87	<0.05	2.25	<0.001	-12.16	<0.05	P/A	-	0.66	<0.01	-0.11	<0.001	(i)
	Epiafzelechin-epicatechin 3,3'-digallate	887.148	6.83	M-H2O-H	1.73042	**8.41**	<0.05	**3.05**	<0.001	0.5	<0.05	-8.85	<0.001	1.93	<0.01	15.42	<0.001	(iii)
	Diosmetin 7-O-beta-D-glucuronopyranoside	476.095476104/ 1048.37584	2.99	M-H2O-H	1.07112	-0.13	<0.05	-0.74	<0.001	0.93	<0.001	0.12	<0.01	**13.17**	<0.001	**17.78**	<0.001	(iii)
	Kaempferol 3-β-d-glucopyranoside	448.1006	5.53	M-H, M+Na-2H	1.53710	**5.19**	<0.001	0.08	<0.001	**-1.7**	<0.05	**-3.11**	<0.001	0.57	<0.05	20.07	<0.05	(i)
	Tricin 7-glucuronide	1034.217561	3.83	M-2H, M-H	1.29161	2.4	<0.001	-0.81	<0.001	-0.47	<0.05	-0.76	<0.01	16.17	<0.001	0.28	<0.05	(iii)
	Prunin 6"-p-coumarate	580.1580764	2.97	M-H2O-H	1.07112	-1.06	<0.01	-2	<0.001	-0.14	<0.05	0.17	<0.05	18.12	<0.01	18.98	<0.001	(i)
	Quercetin 3-glucoside	934.2379024	4	M-H	1.55270	**4.34**	<0.001	**2.02**	<0.001	0.2	<0.05	0.38	<0.05	0.25	<0.05	-0.72	<0.05	(iii)
	Quercetin -(2"-galloyl-α-L-arabinopyranoside)	586.09587	4.25	M+FA-H	1.73065	**15.61**	<0.001	**3.11**	<0.001	-0.66	<0.05	-0.42	<0.05	0.96	<0.05	0.94	<0.05	(iii)
	Catechin	289.0741	2.38	M-H	1.46300	**1.14**	<0.01	**0.99**	<0.001	1.04	<0.05	0.91	<0.001	1.05	<0.05	1.54	<0.05	(iii)
	Epigallocatechin gallate	457.0828	2.99	M-H	1.07112	**1**	<0.01	**0.96**	<0.001	**1.18**	<0.05	**1.02**	<0.001	**0.49**	<0.05	**0.81**	<0.05	(iii)
	Kaempferol 3-sophoroside 7-glucuronide	786.1854729	2.15	M+FA-H	1.19289	**-15.21**	<0.001	**-0.41**	<0.001	-2.48	<0.01	0.96	<0.01	-0.38	<0.05	-0.35	<0.05	(iii)
	Quercetin-3-sulfate	381.9994672	4.5	M+Na-2H	1.07434	-1.43	<0.001	-0.28	<0.001	0.28	<0.05	-0.19	NS	-1.34	<0.05	-17.02	<0.05	(ii)
	(-)-Epigallocatechin 3,3'-di-gallate	610.095870034/ 631.0705	5.82	M-H/M+Na-2H	1.73042	**3.27**	<0.05	**-2.28**	<0.001	0.15	<0.05	-3.32	<0.001	2.61	<0.01	7.44	<0.05	(iii)
	Prunin 6"-O-gallate	586.1322555	5.69	M-H2O-H	1.41871	**3.99**	<0.01	**1.51**	<0.001	**-3.23**	<0.05	**-1.43**	<0.001	-0.35	<0.05	-	-	(iii)

	Gallocatechin	306.0739528	2.04	M-H2O-H	1.41323	**17.14**	<0.001	**0.27**	<0.001	-1.87	<0.001	-0.07	NS	1.28	<0.001	-0.52	<0.05	(i)
	Malvidin	330.047/ 366.050630541	0.81	M-H/M-H2O-H	2.02670	0.42	<0.05	0.07	<0.001	0.06	<0.05	0.46	<0.01	1.56	<0.05	-	-	(iii)
	Epicatechin	207.0662/ 290.07903818	3.32	M-H2O-H	1.34065	P/A	-	-1.23	<0.001	-0.48	<0.05	4.48	<0.01	-0.81	<0.05	0.37	<0.05	(ii)
	(-)-Epiafzelechin 3-gallate	135.9791	2.31	(M-H2O-H, M-H)	1.77701	2.16	<0.01	0.19	<0.001	-0.81	<0.05	-0.23	<0.01	3.64	<0.01	6.13	<0.01	(iii)
	Licorice glycoside A	726.2159852	7.42	M-H	1.53554	-1.55	<0.05	-6.23	<0.001	**2.96**	<0.05	**1.09**	<0.05	0.32	<0.05	-22.17	<0.05	(iii)
	Paratocarpin A	388.1674593	3.44	M-H	1.93691	0.89	<0.01	-0.42	<0.001	-0.69	<0.05	-0.69	<0.001	15.34	<0.001	21.02	<0.001	(i)
Chalcone	4'-Methoxychalcone	534.1502	2.62	/M-H2O-H	1.66973	**-4.42**	<0.05	**-8.48**	<0.001	**-3.82**	<0.001	**-3.71**	<0.001	-0.7	<0.05	-0.69	<0.05	(i)
	4,2',4',alpha-tetrahydroxydihydrochalcone	274.0841236	4.13	M+Na-2H	1.21483	**-6.9**	<0.001	**-20.32**	<0.001	-0.5	<0.001	0.47	<0.01	-0.39	<0.05	0.21	<0.05	(i)
Benzenoids	Gallic acid	170.0215233	1.43	M-H2O-H	1.14956	**8.36**	<0.05	**-2.57**	<0.001	-0.94	<0.05	-0.33	<0.001	5.86	<0.05	-1.43	<0.05	(i)
	M-trigallic acid	474.0434405	3.32	M+Na-2H/M-H2O-H	1.05172	**2.33**	<0.001	**1.12**	<0.001	-15.07	<0.001	P/A	-	0.77	<0.05	1.76	<0.05	(i)
	2,4-dihydroxybenzoic acid	154.0266087	3.31	M-H2O-H	1.34065	0.28	<0.05	0.2	<0.001	0.32	<0.05	0.55	<0.001	-17.42	<0.001	-14.81	<0.05	(ii)
	Catechol	110.0367794	3.06	M-H2O-H	1.33244	0.09	<0.05	-0.96	<0.001	**-10.96**	<0.001	0.68	<0.001	P/A	-	0.56	<0.05	(i)
Organic Acids	Quinic acid	192.0633881	0.81	M-H2O-H	1.32319	1.23	<0.001	1.04	<0.001	-0.46	<0.05	1.06	<0.001	**14.48**	<0.001	**2.08**	<0.05	(i)
	Shikimic acid	174.0528234	3.43	M-H	1.33649	-0.87	<0.01	2.6	<0.001	0.73	<0.001	2.72	<0.001	**12.95**	<0.01	**13.99**	<0.05	(i)

‘P/A’ means a metabolite is detected in –P but absent in +P, or vice versa.

[a]Identification Category
(i) Actual Mass (AM), Retention Time and Standard
(ii) AM and RT
(iii) AM and Isotopic Distribution (ID)

Table S3. Transcription factor primer sequences for *cv. assamica* (Fengqing) and *cv. sinensis* (Longjing-43) for qRT-PCR analysis.

**Transcription Factor**	**Cultivar**	**Gene Identifier**	**Forward primer (5'>3')**	**Reverse Primer (5'>3')**
PHR1 (phospahte starvation response 1)	***cv. sinensis***	CL15435Contig1	ATCTGCACAATGAACAGCACCAT	GCTCCAAAGAGGCTTAGGATAGA
	cv. assamica	CL69205Contig1	CTGCCCAGAATCGGACAATAAGA	AACGCAAACTGAGAATCCTGAAATG
PHO1 (phosphate signal transduction 1)	***cv. sinensis***	CL27009Contig1	TCGAGTCCTGGTAGGAAGAACCT	GTTGATTATTGGCAACTCAAGAAAG
	cv. assamica	CL39177Contig1	ATCATCCCATACAAGTACAAGAATC	CTAAATGTCCAAACTCCACCTAA
SPX2 (Pi transport, Pi stress, sensing and signalling)	***cv. sinensis***	CL117Contig2	CAGGGTAGCAGAGGCTCGGGATT	AGCACCGCTTCGCTTGTCGTATT
	cv. assamica	CL58020Contig1	GCGGTGGCAACGAGAACTCACTC	CTTCTTCGCTCGCCTTTCATTCA

Table S4. Primer sequences for the gene transcripts of *cv. assamica* (Fengqing) cultivar of selected pathways for qRT-PCR analysis.

**Pathway**	**Reaction, Gene and Enzymatic activity**	**Gene Identifier *cv. assamica***	**Forward primer (5'>3')**	**Reverse Primer (5'>3')**
Valine, Leucine and Isoleucine Biosynthesis	Branch Chain Amino Transferease 1 (2.6.1.42, *BCAT1*)	CL60167Contig1	GCCTTTGACATCCTTTGATTCTG	GGAGCTTATCTTGCCTTCTTTCG
Phenylalanine,Tyrosine and Tryptophan biosynthesis	tryptophan synthase alpha chain (*TSA1*, 4.2.1.20)	CL76112Contig1	ACATTTATCGGAAGGTCGCATTT	TAACAAGTAAGCCTGTGGCAGTT
	arogenate/ prephenate dehydrase (*ADT1*, 4.2.1.51/4.2.1.91)	CL49595Contig1	CAGCAGAACAGTGGCTATGGAAT	TGAATGGAAGTAACCTCGAAGAAT
Methionine Biosynthesis	methyltransferase (2.1.1.14, *MS2*)	CL27145Contig1	CTCCAGTCACAAGCGTGTAGCCT	GTCCTCGTTCTGGTCTCATCTTCTTTC
Alanine, asparate and glutamate biosynthesis	Omega-amidase, chloroplast (3.5.1.111, *NPL3*)	CL69393Contig1	ACTATTCCATATTTCAGGCAAGAC	CCACTACCACCACCTATCCATCT
	glutamate synthase (1.4.1.13/1.4.1.14, *GLT1*)	CL40912Contig1	TGTATGAGGAAGAAACCAGCAAC	TAAAGACGGAGTATCCTAAAGGT
Butanoate metabolism	acyl-COA synthetase (6.2.1.2, *ACS*)	CL45357Contig1	GCATAGGTGTCGGAGTCTTTGAG	GGAACCACTTGAGCGACCTTACT
Arginine and Proline Biosynthesis	prolyl 4-hydroxylase (1.14.11.2, *P4H*)	CL35020Contig1	CAGTTTGCCAAATGAACTATCAAG	TATGGTAGGCTCTGAAGGTGTAG
Flavonoid Biosynthesis	anthocyanidin reductase (1.3.1.77, *ANR*)	CL2560Contig1	GGACTTCTTAGGTGGGATTTCTA	AATCATCTGGCTGGCTTACTACA
	leucoanthocyanidin dioxygenase (1.4.11.19, *LDOX*)	CL59191Contig1	TCCATACTCTTCTATCACTTCCCTAA	AGTAATCCGTGTTCAATCCTTGT
Flavone and Flavonol Biosynthesis	glucosyltransferases (2.1.4.81, *UGT75L12*)	CL68486Contig1	ATCTACTGTTATGACATGGGCTTTA	ATCTTACAATGGTGATTTGAGGG
	flavonol 3-O-glucosyltransferase (2.4.1.91, *UGT78D2*)	CL4384Contig1	TAATCATCGGAAGACAAGCACAT	AAGGGTTGATAGTGAGGAGTTGG
	flavonol-3-O-glucoside L-rhamnosyltransferase (2.4.1.159, *UGT78D1*)	CL38490Contig1	GGTAGGTGAGTTGTGAAATCGCTGAA	GCTGGGCTCTTGGATGCTTCTTC

Table S5. Primer sequences for the gene transcripts of *cv. sinensis* (Longjing-43) cultivar of selected pathways and the reference was used for qRT-PCR analysis.

**Pathway**	**Reaction, Gene and Enzymatic activity**	**Gene Identifier *cv. sinensis***	**Forward primer (5'>3')**	**Reverse Primer (5'>3')**
Valine, Leucine and Isoleucine Biosynthesis	Branch Chain Amino Transferease 1 (2.6.1.42, *BCAT1*)	CL23657Contig1	TATGTTGTTGAGGAGGTTGTTGT	GAAAGACTATCATCCCAGCCAGA
Phenylalanine, Tyrosine and Tryptophan biosynthesis	tryptophan synthase alpha chain (*TSA1*, 4.2.1.20)	CL5201Contig2	GCTCGTGTATCTGTAAGCGGAAAG	CACTATGGCACTACCAACAATCA
	arogenate/ prephenate dehydrase (*ADT1*, 4.2.1.51/4.2.1.91)	CL9637Contig1	CAACTGGTCGTTTGTCCGTAATG	CTTGCTCAGACTTCCTAAGAGGC
Methionine Biosynthesis	methyltransferase (2.1.1.14, *MS2*)	CL16257Contig1	ATATGCCATGACTGGAGCGATGA	GGTATTGAAAGCGCAGCACATCA
Alanine, asparate and glutamate biosynthesis	Omega-amidase, chloroplast (3.5.1.111, *NPL3*)	CL22649Contig1	ACTAATCAGGTCACTACGGTCAA	AGGTCAGGGAACTTCTAGGGTAT
	glutamate synthase (1.4.1.13/1.4.1.14, *GLT1*)	CL21Contig13	ACATCACGCATAGCATCCATCTC	GTTCACTGTAATTGTGGTTCCCT
Butanoate metabolism	acyl-COA synthetase (6.2.1.2, *ACS*)	CL263Contig1	TGCTGCGTCTTCACTCACTGTTT	TCCTTGTAAGCCTTATGCCATCC
Arginine and Proline Biosynthesis	prolyl 4-hydroxylase (1.14.11.2, *P4H*)	CL32565Contig1	AAATCGGACTGGAGGGTGCTGTT	TGTCTTCGTTGCTGAGTTGAGGG
Flavonoid Biosynthesis	anthocyanidin reductase (1.3.1.77, *ANR*)	CL16739Contig1	ATCAAGGACATGGCAAGACCAAT	GAGCAGACCTCACCGATGAACAG
	leucoanthocyanidin dioxygenase (1.4.11.19, *LDOX*)	CL12667Contig1	TGAGCCGCCAAAGGAGAAGATTA	CAGGCAAAGGACAAGCACTGAGACA
Flavone and Flavonol Biosynthesis	glucosyltransferase (2.4.1.81, *UGT75L12*)	CL10868Contig1	AGAATAACTCGCCGCACAACCTG	CCATCCTCAAGACCATCAAACCC
	flavonol 3-O-glucosyltransferase (2.4.1.91, *UGT78D2*)	CL11167Contig1	CAAACACCTTTACCCAGTCCACC	GCATCTGAGACCTCGAAATACCA
	flavonol-3-O-glucoside L-rhamnosyltransferase (2.4.1.159, *UGT78D1*)	CL72084Contig1	TTCGCAGTCATCAAGATCGTGTC	TTTGTTCAACGCAACTTCCGTCA
